# Effect of Intravenous Aminophylline on Hemodynamics and Recovery of Patients Undergoing Functional Endoscopic Sinus Surgery Under Dexmedetomidine Hypotensive Anesthesia: A Randomized Controlled Study

**DOI:** 10.5812/aapm-141669

**Published:** 2023-12-29

**Authors:** Osama Mohammed Rehab, Doha Mohammed Bakr, Osama Abdelmoneam Algazzar, Islam Morsy

**Affiliations:** 1 Anesthesiology, Surgical Intensive Care and Pain Management Department, Faculty of Medicine, Tanta University, Tanta, Egypt; 2 Anesthesiology, Surgical Intensive Care and Pain Management Department, Faculty of Medicine, Helwan University, Helwan, Egypt; 3 Anesthesia Department, Mediclinic Middle East, United Arab Emirates

**Keywords:** Aminophylline, Recovery, Hemodynamics, Endoscopic Sinus Surgery, Dexmedetomidine

## Abstract

**Background:**

The sympatholytic property of dexmedetomidine (DEX) makes it suitable as a hypotensive drug during functional endoscopic sinus surgery (FESS); however, delayed emergence from anesthesia and high postoperative sedation have been reported.

**Objectives:**

Delayed emergence from anesthesia and high postoperative sedation are associated with a prolonged length of stay in the operating room and the postanesthesia care unit (PACU), which increases health care costs. This study aimed to overcome the negative impact of DEX on recovery by using aminophylline.

**Methods:**

This randomized, double-blind, placebo-controlled study was conducted on 52 patients planned for elective FESS under general anesthesia with DEX infusion for controlled hypotension during surgery. Patients were equally divided into 2 groups. The aminophylline group received 4 mg/kg aminophylline diluted in 50 mL saline 0.9% over 30 minutes after positioning in a 20-degree reverse Trendelenburg position. The control group received 50 mL saline 0.9% with a similar volume and period as the aminophylline group.

**Results:**

The extubation time was significantly shorter in the aminophylline group (6.5 (5.25 - 7.75) minutes) than in the control group (9 (7.25 - 10) minutes) (P-value < 0.001). The PACU discharge time was significantly shorter in the aminophylline group (15 (10 - 20) minutes) compared to the control group (20 (15 - 28.75) minutes) (P-value = 0.036). Intraoperative heart rate and mean arterial pressure were nonsignificantly different between the 2 groups. Ramsay sedation score measurements at 15 min, 30 min, and 60 min after extubation were significantly lower in the aminophylline than in the control group (P-value < 0.05). Complications were nonsignificantly different between the 2 groups.

**Conclusions:**

Intraoperative aminophylline infusion enhances the recovery of patients undergoing FESS under DEX hypotensive anesthesia without intraoperative hemodynamic alterations and decreases their postoperative sedation without significant postoperative side effects.

## 1. Background

Functional endoscopic sinus surgery (FESS) is a crucial therapeutic approach for nasal sinus diseases (1, 2). Impairment of operative field visibility by bleeding is an essential concern in FESS, and serious problems have been documented for FESS due to reduced visibility (3, 4). As a result, controlled hypotensive anesthesia is essential in FESS as it decreases intraoperative blood loss, creating an optimal field for surgery (5, 6).

Dexmedetomidine (DEX) is an α2 adrenergic receptor agonist with high selectivity. It has a sympatholytic property as it reduces norepinephrine release. This lowers the heart rate (HR), arterial blood pressure, and cardiac output in a dose-dependent manner and makes it useful as a sedative, analgesic, and anesthetic-sparing agent (7-9). These beneficial effects of DEX made it a desirable option for establishing hypotensive anesthesia during FESS (10-12). However, most studies that used DEX during FESS reported delayed emergence from anesthesia and higher postoperative sedation with or without delay in a postanesthesia care unit (PACU) discharge time (13-16).

Aminophylline exerts its effect by either inhibiting phosphodiesterase or blocking the central adenosine receptors and attenuating gamma-aminobutyric acid neurotransmission (17). Thus, it can reduce the level of sedation and hypnosis and shorten the recovery time (18). It has been shown in previous studies that aminophylline could enhance the recovery of patients who received propofol anesthesia or inhalational anesthesia (18-21) and antagonized different drugs, such as benzodiazepines, opioids, barbiturates, and inhalational anesthetics (22-27). However, no study has evaluated its effect on patients receiving intraoperative DEX.

## 2. Objectives

We conducted this study to assess the impact of intravenous aminophylline on the hemodynamics and recovery of patients receiving intraoperative DEX infusion during FESS. The time to extubation was our primary outcome, and secondary outcomes included intraoperative hemodynamics, PACU discharge time, postoperative sedation scores, and side effects.

## 3. Methods

This randomized, double-blind, placebo-controlled study was conducted at Tanta University Hospitals from February 25, 2023, to August 2023 on 52 patients of both sexes, aged 18 to 50 years, with the American Society of Anesthesiology (ASA) physical status ≤ II, and planned for FESS under general anesthesia with DEX infusion for controlled hypotension during surgery. Enrollment of the patients was done after approval by the Institutional Ethical Committee of Tanta University (approval code 36264PR58/1/23) and registration in the clinical trials registry (ID: NCT05738135) by the primary investigator on February 21, 2023. The protocol was in accordance with the Declaration of Helsinki guidelines. All the participants signed informed consent forms. Patients with a body mass index ≥ 30 kg/m^2^, allergy to aminophylline, central nervous system diseases, hypertension, arrhythmias, cerebrovascular diseases, convulsions, renal impairment, or hepatic dysfunction were excluded. Pregnant or lactating females, patients with recurrent sinus surgery, addiction to opioids, excessive coffee intake (greater than 2 cups each day), and conditions requiring beta 2 agonists, tranquilizers, or antidepressant medications were excluded.

 Patients were randomly assigned into 2 equal groups (26 patients in each group), utilizing computer-generated numbers and sealed opaque envelopes. Patients in the aminophylline group (Group 1) received 4 mg/kg aminophylline (Etaphylline™, Memphis for Pharmaceutical & Chemical Industries, Egypt) diluted in 50 mL saline 0.9% intravenously (IV) over 30 minutes after positioning in a 20-degree reverse Trendelenburg position. Patients in the saline group (Group 2) were given an equivalent volume of isotonic saline for the same duration.

Cases were unaware of the type of drug received. An independent anesthetist not involved in the study prepared the study solutions in identical syringes; both aminophylline and saline were clear and transparent solutions prepared in an equal volume of 50 mL. The study solutions were administered over 30 minutes to avoid any hemodynamic changes that might occur with the rapid injection of aminophylline, which could affect its blinding, to minimize the potential biases. Also, a single surgeon performed all the procedures, and a single anesthesiologist performed the anesthetic management. Another anesthesiologist recorded the intraoperative data and assessed the postoperative outcomes. Both anesthesiologists and the surgeon were unaware of the patients’ group assignment.

Before induction of anesthesia, a loading dose of 1 microgram (µg)/kg DEX diluted in 50 mL 0.9% saline was given as an infusion over 15 min to all patients while they were monitored by non-invasive arterial pressure, pulse oximetry, and electrocardiogram (ECG). A temperature probe and capnogram were added for intraoperative monitoring. For general anesthesia induction, 2 µg/kg fentanyl, 2 mg/kg propofol, and 0.5 mg/kg atracurium were used. After intubation, a pack in the oropharynx was inserted, and a titrated continuous infusion of DEX at a rate of 0.4 - 0.8 µg/kg/h was applied to all the patients to keep the intraoperative mean arterial pressure (MAP) values at 60 - 65 mmHg and keep HR values below baseline values. This infusion would be temporarily discontinued if MAP values decreased below 60 mmHg and would be stopped 10 minutes before the end of the surgery. The end-tidal carbon dioxide was kept between 35 and 40 mmHg by manipulating the ventilator settings.

Maintenance of anesthesia was done by sevoflurane 2% in air/oxygen. Top-up atracurium doses (0.1 mg/kg) were administered to maintain the neuromuscular block as needed. Intraoperative 4 mg dexamethasone IV and 1 g paracetamol IV infusion were given to all patients. A 20-degree reverse Trendelenburg position was applied to all the patients before the start of the surgical procedure. After positioning, the study solutions were given, as mentioned, only to cases that had MAP and HR values below baseline values.

Approximately 15 minutes after the completion of the study drug infusion, the surgeon (the only person who performed all the procedures) assessed the operative field quality depending on Fromme et al.’s category scale (28, 29).

In both groups, intraoperative HR and MAP were recorded at baseline, after the loading dose of DEX, after intubation, every 5 minutes during the study drug infusion, and then every 15 minutes until the end of surgery. Increased HR above the baseline value or MAP above the planned target (65 mmHg) was treated by raising the volatile anesthetic’s concentration to 1.3 of its minimum alveolar concentration (MAC) and the infusion rate of DEX (up to 0.8 µg/kg/h). If there was no response within 5 minutes, 0.5 µg/kg of fentanyl would be given. If MAP values decreased below 60 mmHg, DEX infusion would be temporarily discontinued, sevoflurane concentration would decrease to 0.7 of its MAC, and ephedrine (6 mg IV bolus) would be given. The HR value of less than 50 beats/min was managed by giving atropine 0.6 mg IV and temporarily discontinuing the DEX infusion.

At the end of the surgery, the neuromuscular blockade was reversed by neostigmine and atropine. Extubation was performed when the extubation criteria were met after the removal of the pack. The extubation time, our primary outcome, i.e., the duration between the closure of inhalational anesthetic to safe tracheal extubation, was recorded. Then, the patients were transferred to PACU. Postoperative sedation was assessed at 15, 30, 60, and 120 minutes after extubation by the Ramsay sedation score (30). The modified Aldrete score (31) was used to assess the criteria needed to discharge our patients from the PACU, and the time required to achieve a score ≥ 9 was recorded. Postoperative side effects, such as nausea, vomiting, shivering, arrhythmia, and lightheadedness, were recorded.

### 3.1. Sample Size Justification

The sample size was estimated using G*Power v. 3.1.9.2 (Universitat Kiel, Germany). A previous study showed that the mean value of extubation time in the aminophylline group decreased by 2.5 minutes (32), this yielded a 0.841 effect size. With a 2-tailed *t*-test, 80% power, and α of 0.05, a minimum sample size of 24 patients in each group was required; as 10% was added for dropouts, 52 patients were eventually recruited.

### 3.2. Statistical Analysis

SPSS v. 27 (IBM, Armonk, NY, USA) was used to analyze our data. Histograms and the Shapiro-Wilks test analyzed the data distribution normality. Unpaired Student’s t-tests analyzed quantitative parametric data expressed as mean ± standard deviation (SD). The Mann-Whitney U test analyzed quantitative, nonparametric data described as the median and interquartile range (IQR). The chi-square or Fisher’s exact test analyzed the qualitative variables described as frequency (%). A 2-tailed P-value < 0.05 was considered significant.

## 4. Results

**Figure 1.**
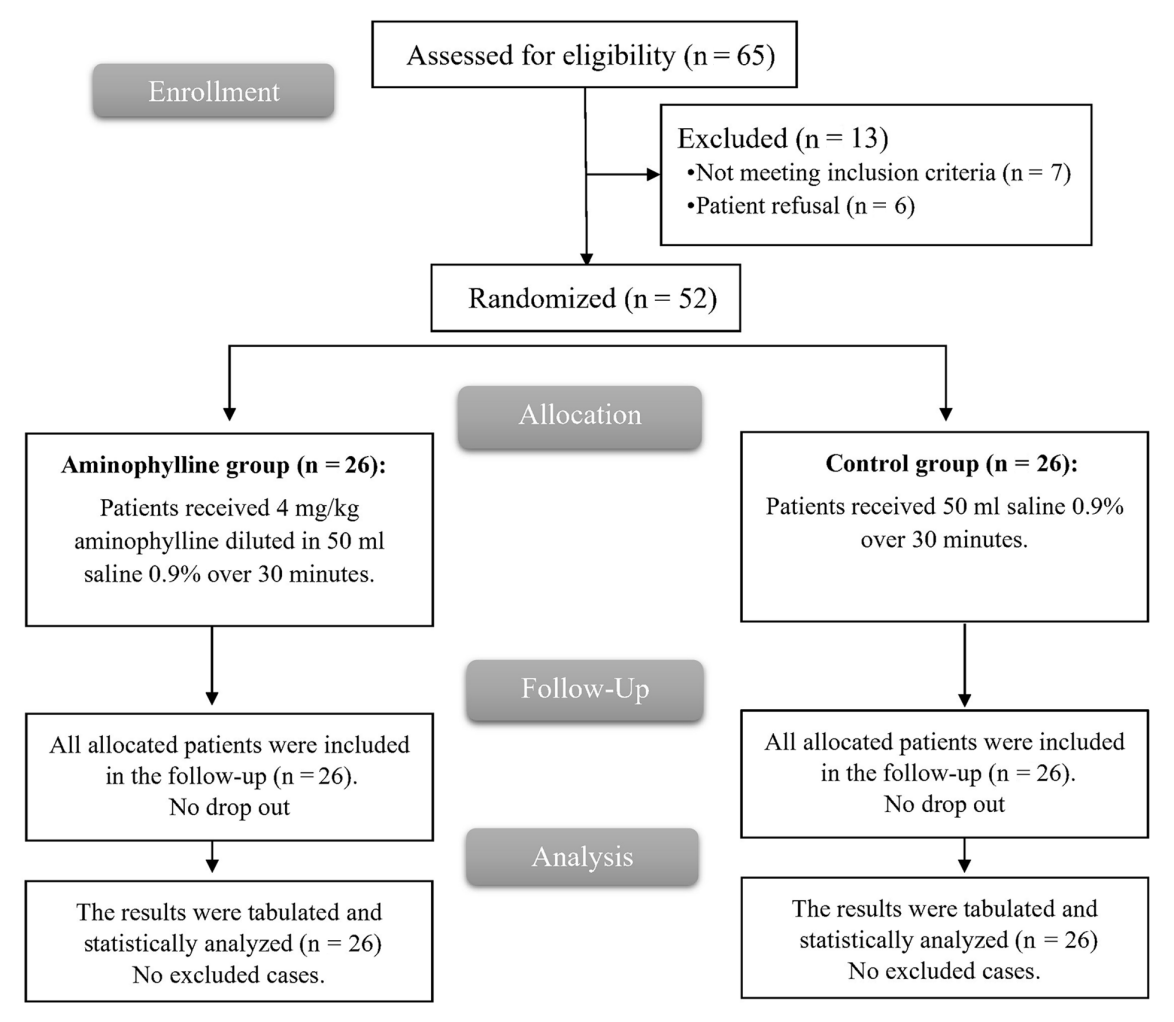
CONSORT flowchart of the enrolled patients

In this study, the eligibility of 65 patients was evaluated; 7 patients were not eligible, and 6 declined to participate in the trial. The rest of the patients were randomized into 2 groups (26 patients each). The HR and MAP values were lower than the baseline values in all cases at the time of solution administration, so all the patients were given the study drug, followed up, and statistically analyzed (Figure 1). 

Demographic data, duration of surgery, and average category scale were nonsignificantly different between the 2 groups (Table 1). 

**Table 1.** Demographic Data, Duration of Surgery, and Average Category Scale of the Studied Groups ^a^

Variables	Aminophylline Group (N = 26)	Control Group (N = 26)	P-Value
**Age (y)**	28.77 ± 6.95	30.5 ± 6.33	0.353
**Sex**			
Male	16 (61.54)	14 (53.85)	0.575
Female	10 (38.46)	12 (46.15)	
**ASA physical status**			
I	22 (84.62)	19 (73.08)	0.308
II	4 (15.38)	7 (26.92)	
**Weight (kg)**	65.77 ± 7.7	68.5 ± 9.84	0.270
**Duration of surgery (min)**	98.65 ± 14.73	91.54 ± 18.59	0.132
**Average category scale **	2 (2 - 3)	2 (1 - 2.75)	0.470

Abbreviation: ASA, American Society of Anesthesiologists.

^a^ Data are presented as mean ± SD or frequency (%) or median (IQR).

**Figure 2.**
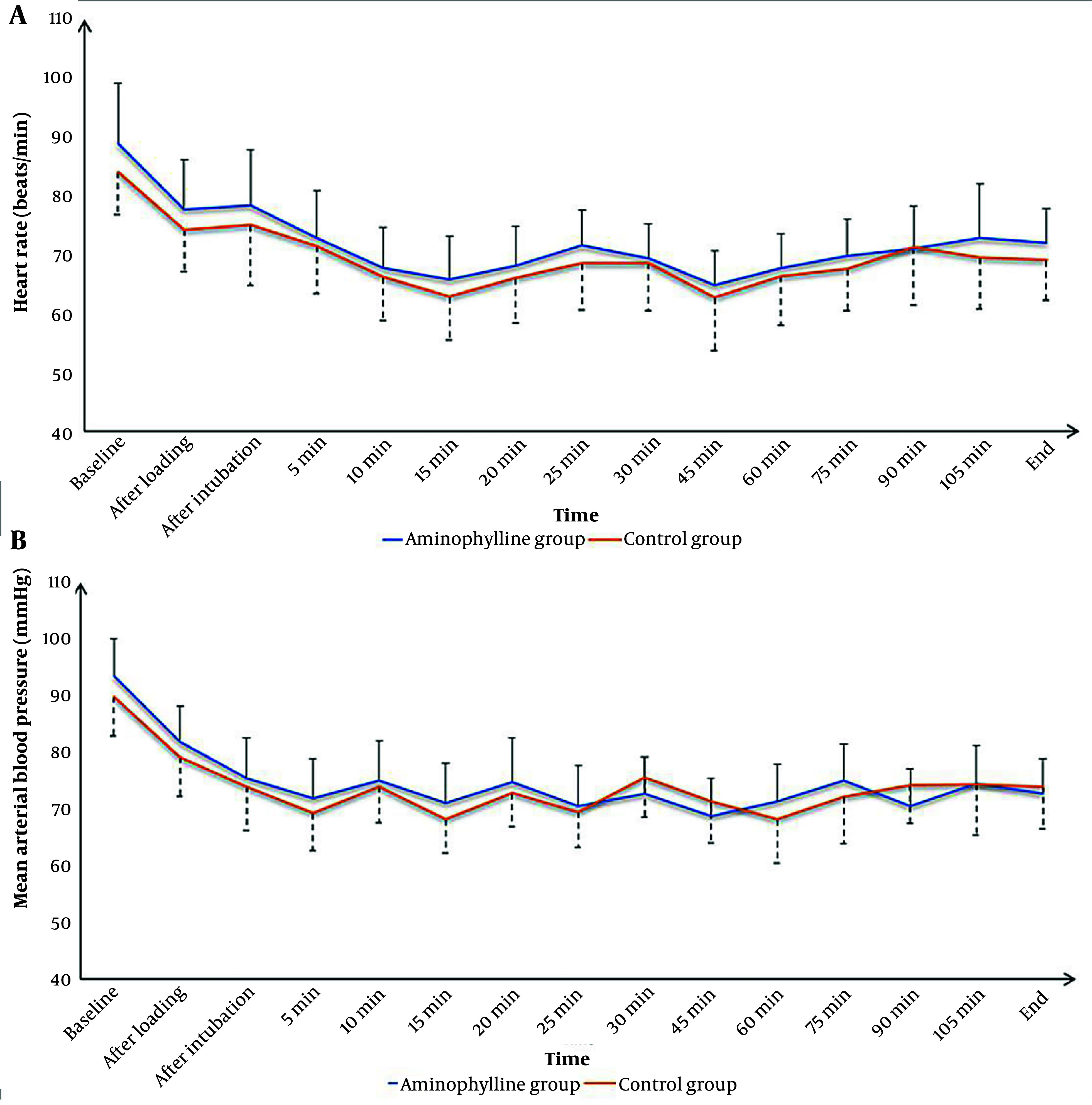
Intraoperative heart rate (A); and mean arterial blood pressure (B) changes of the studied groups

Intraoperative HR and MAP values at baseline, after the loading dose of DEX, after intubation, at 5 min, 10 min, 15 min, 20 min, 25 min, 30 min, 45 min, 60 min, 75 min, 90 min, 105 min, and at the end of the surgery were nonsignificantly different between the 2 groups (Figure 2). 

Only 1 case in the aminophylline group required a single bolus dose of 0.5 µg/kg fentanyl, and no case in either group required a vasodilator drug.

The Ramsay sedation score measurements at 15 min, 30 min, and 60 min after extubation were significantly lower in the aminophylline than the control group (P-value<0.05) and were nonsignificantly different at 120 min after extubation between the 2 groups (Table 2). 

**Table 2.** Ramsay Sedation Score Measurements after Extubation of the Studied Groups ^a^

Variables	Aminophylline Group (N = 26)	Control Group (N = 26)	P-Value
**15 min**	2.88 ± 0.71	3.31 ± 0.68	0.033 ^b^
**30 min**	2.46 ± 0.51	2.85± 0.67	0.024 ^b^
**60 min**	2.04 ± 0.34	2.35 ± 0.49	0.011 ^b^
**120 min**	1.73 ± 0.45	1.81 ± 0.49	0.560

^a^ Data are presented as mean ± SD.

^b^ Significant when P-value ≤ 0.05.

The median (IQR) extubation time was significantly shorter in the aminophylline group (6.5 (5.25 - 7.75) minutes) than in the control group (9 (7.25 - 10) minutes) (P-value < 0.001). The median (IQR) PACU discharge time was significantly shorter in the aminophylline group (15 (10 - 20) minutes) compared to the control group (20 (15 - 28.75) minutes) (P-value 0.036). Complications (hypotension, bradycardia, postoperative nausea and vomiting (PONV), headache, and lightheadedness) differed insignificantly between the 2 groups (Table 3). 

**Table 3.** Extubation Time, Time to Postanesthesia Care Unit Discharge, and Side Effects of the Studied Groups ^a^

Variables	Aminophylline Group (N = 26)	Control Group (N = 26)	P-Value
**Extubation time (min)**	6.5 (5.25 - 7.75)	9 (7.25 - 10)	<0.001*
**Time to PACU discharge (min)**	15 (10 - 20)	20 (15 - 28.75)	0.036*
**Side Effects**			
**Hypotension**	2 (7.69)	4 (15.38)	0.668
**Bradycardia**	1 (3.85)	3 (11.54)	0.609
**PONV**	3 (11.54)	1 (3.85)	0.609
**Headache**	2 (7.69)	1 (3.85)	1
**Lightheadedness**	2 (7.69)	0 (0)	0.490
**Arrhythmia**	0(0)	0 (0)	---

Abbreviations: PONV: postoperative nausea and vomiting; PACU: postanesthesia care unit

^a^ Data are presented as median (IQR) or frequency (%).

## 5. Discussion

The sympatholytic properties of DEX make it suitable as a hypotensive drug during FESS; however, delayed emergence from anesthesia was reported. Therefore, we conducted our study to overcome this negative impact of DEX on recovery by using aminophylline.

The results of our study showed that the recovery profile was enhanced with aminophylline, as evidenced by a shorter extubation time, PACU discharge time, and reduced postoperative sedation. Aminophylline has a neuronal excitability effect as it blocks the adenosine receptors mediating hypnosis and inhibits gamma-aminobutyric acid in the central nervous system (17, 33). This aminophylline effect could attenuate the central α2 adrenergic receptor-mediated sedative effects of DEX.

Regarding the recovery profile, our results were consistent with those of previous studies that reported an enhancing effect of intraoperative aminophylline infusion on the recovery of patients undergoing different surgeries in which various anesthetic modalities were utilized.

A recent study assessed the impact of 3 mg/kg IV aminophylline on the recovery from isoflurane general anesthesia after abdominal hysterectomy (34), and its results revealed that aminophylline led to a significant reduction in extubation time and PACU discharge time.

Also, Kadhim evaluated the role of aminophylline on the recovery of patients undergoing orthopedic procedures using total intravenous anesthesia (35). Patients received either 4 mg/kg aminophylline or saline once the procedure was completed. They reported significantly shorter extubation time in the aminophylline group compared to the saline group.

Furthermore, El Tahan evaluated the effect of various dosages of aminophylline on cognitive recovery from sevoflurane-fentanyl anesthesia (26). Cases were given aminophylline of 2, 3, 4, or 5 mg/kg or saline at the end of anesthesia. The aminophylline groups recovered more quickly from anesthesia than the saline group. This was shown by shorter extubation and PACU discharge times.

Moreover, Turan et al. demonstrated that aminophylline increased the propofol dose required to produce unconsciousness and reduced the anesthetic depth, with a shorter time to return to consciousness for the aminophylline group than the saline group (36).

Also, aminophylline’s role in recovery from sevoflurane anesthesia was investigated by Turan et al. (19). Cases were given either 5 mg/kg aminophylline IV or saline 0.9% after sevoflurane closure, and they reported that extubation time was decreased significantly in the aminophylline group.

Also, Jeon et al. demonstrated that aminophylline’s arousal effect accelerated propofol fentanyl-sevoflurane anesthesia recovery and lowered sedation depth without affecting hemodynamics (37).

Moreover, Ghaffaripour et al. studied aminophylline’s impact on patients’ recovery from total intravenous anesthesia (32). They concluded that injecting aminophylline at emergence significantly shortened the recovery time in their patients.

Besides, the results of Kasim et al. revealed that pretreatment with aminophylline led to a significant reduction in extubation time with no significant effect on PACU discharge time in pelvic-abdominal surgeries (27).

These observations during recovery time support the idea that aminophylline can counteract the sedative-hypnotic effects of general anesthetics even with intraoperative DEX infusion.

Our concern about using aminophylline in our study was that it might antagonize the beneficial hemodynamic effects of DEX. Thus, we used 4 mg/kg as an infusion for 30 minutes, as it was known that this long infusion period would not affect HR or blood pressure (38, 39).

Our results demonstrated that aminophylline did not affect the intraoperative hemodynamics of either HR or MAP without a significant difference between it and placebo. These results are consistent with those of previous studies that used aminophylline in other types of surgeries and reported insignificant differences between aminophylline and placebo regarding hemodynamics (26, 27, 37).

Hüpfl et al. demonstrated that 3 mg/kg aminophylline was injected IV over 1 minute, and no patient experienced arrhythmia or tachycardia following aminophylline injection. Furthermore, HR and MAP values were comparable between the aminophylline group and the control group (18).

However, Turan et al. reported that aminophylline injection increased HR significantly compared to placebo, with no significant differences in MAP between the 2 groups (19). This rise in HR can be explained by the larger (5 mg/kg) dose of aminophylline used and the timing of its administration after inhalational anesthetic closure, which makes patient recovery a contributing factor in causing tachycardia.

Contrary to our findings, Ghaffaripour et al. divided their patients to receive either saline or aminophylline 4mg/kg over 2 minutes at the end of the surgery (32). Their results showed that aminophylline injection significantly increased MAP and HR values compared to placebo. A shorter infusion period (2 minutes) and delivery time at patient recovery may explain this difference.

Moreover, injecting aminophylline (5 mg/kg) over 1 minute caused a significant increase in HR at 2 to 6 minutes after injection compared to placebo in the study by Turan (20).

Regarding side effects, our results revealed insignificant differences between the 2 groups. Previous studies, such as Hupfl et al. (18), Kasim et al. (27), and Moradi Farsani et al. (40), reported the safety of aminophylline as there were no reported side effects. Also, Djaladat et al. reported self-limited headache and dizziness in 7.1% of patients who received aminophylline for renal colic (41).

However, in partial agreement with our results, El Tahan reported that nausea, vomiting, and tremors incidences were comparable between the aminophylline and placebo groups. Meanwhile, agitation and shivering occurred more frequently in the placebo group (26).

### 5.1. Limitation of Study

Although the sedation scores and PACU discharge time were statistically significantly decreased in the aminophylline group, the difference between the 2 groups may be of little clinical significance. A higher aminophylline dose (6 mg/kg), different surgical procedures, or a larger sample size, including elderly patients, may be considered in further studies to confirm a clinical benefit. Another limitation was the single evaluation of the average category scale 15 minutes after the completion of the study drug infusion. However, HR and MAP values, essential factors in determining the optimal surgical field, were comparable between the 2 groups throughout the procedure.

### 5.2. Conclusions

Intraoperative aminophylline infusion enhances the recovery of patients undergoing FESS under DEX hypotensive anesthesia without intraoperative hemodynamic alterations and decreases their postoperative sedation without significant postoperative side effects.

## Data Availability

The dataset presented in the study is available on request from the corresponding author during submission or after publication.

## Clinical Trial Registration Code:

NCT05738135.
